# Self-Concept as a Mediator of the Relation Between University Students’ Resilience and Academic Achievement

**DOI:** 10.3389/fpsyg.2021.747168

**Published:** 2022-01-04

**Authors:** Inmaculada García-Martínez, José María Augusto-Landa, Rocío Quijano-López, Samuel P. León

**Affiliations:** ^1^Department of Didactics and School Organization, Faculty of Education, University of Granada, Granada, Spain; ^2^Department of Psychology, Faculty of Humanities and Educational Sciences, University of Jaén, Jaén, Spain; ^3^Department of Science Education, Faculty of Humanities and Educational Sciences, University of Jaén, Jaén, Spain; ^4^Department of Pedagogy, Faculty of Humanities and Educational Sciences, University of Jaén, Jaén, Spain

**Keywords:** self-concept, resilience, emotional intelligence, academic achievement, university students

## Abstract

Academic achievement is a factor of interest in both psychology and education. Determining which factors have a negative or positive influence on academic performance has produced different investigations. The present study focuses on analyzing the relationship between resilience, emotional intelligence, self-concept and the academic achievement of university students. For this purpose, different self-report tools were administered to a sample of 1,020 university students from Southern Spain. The Structural Equation-based mediational analysis suggests that there is no direct relationship between resilience and academic achievement, nor between emotional intelligence and academic achievement. Likewise, self-concept is positioned as a mediating factor in the relationship between resilience and academic achievement. The findings indicate that university students who exhibit high levels of resilience tend to cope better with difficult moments and understand and value the effort required and invested in study time. This study supports positive beliefs and behaviors for better academic achievement.

## Introduction

Predicting and explaining academic performance and researching the factors related to students’ academic success are highly important topics in the field of education (Ruban and McCoach, 2005). Academic performance is an important predictor of the future achievements in subsequent educational stages, as well as other important occupational outcomes, such as job performance and salary (Kuncel et al., 2005). University students’ achievement is affected by different factors, such as social, psychological, economic, environmental and individual factors. All of them affect student achievement and they differ among people and among countries. Among these reasons, the present study is focused on psychological factors, in line with previous studies that have attempted to predict the factors involved in student performance from the field of psychology (Wilson and Buttrick, 2016; Asikainen et al., 2020). Therefore, it can be stated that this study is between the fields of education and psychology. An overview of the scientific literature showed studies that have analyzed the relationships between academic achievement and different psychological constructs, such as self-concept (Dweck, 2006; Susperreguy et al., 2018; Wolff et al., 2018; Sewasew and Schroeders, 2019), resilience (Reynoso, 2008; Hudson, 2009; O’Looney, 2010; Wilkinson, 2012) and Emotional Intelligence (EI) (Corcoran and O’Flaherty, 2018; Deighton et al., 2018; Piqueras et al., 2019). However, to the best of our knowledge, no study was found to address the contribution of these constructs jointly in the analysis of academic achievement in university students. This idea implies that the main strength of the current study was the joint consideration of several psychosocial factors (resilience, emotional intelligence and self-concept) in the analysis of the prediction of the academic achievement of university students.

The identification of these associations allows for a more reliable understanding of how different psychosocial factors are related to the academic performance of young university students at the same time. In this sense, this knowledge will provide the basis for the implementation of programs that help to improve emotional intelligence, self-concept or resilience in the university environment. Likewise, and more specifically for instructional processes, it will help teachers to take into account the influence of resilience, self-concept and emotional intelligence when they design and implement their teaching practices.

## Theoretical Framework

### Resilience and Academic Achievement

Resilience is defined as a process, capacity or result of a successful adaptation during and after an exposure to a risk situation (Luthar, 2006). Other authors define it as the “capacity of a dynamic system” to overcome adverse experiences (Masten, 2014). People who competently cope with difficult situations demonstrate the presence of psychological resilience. Resilience may produce a positive chain reaction that leads to fighting adversity and enhancing favorable outcomes (Daniel and Wassell, 2002). This implies a healthy and stable trajectory of functionality, ranging from returning to a balanced state to developing optimal conditions of functioning (Tedeschi and Calhoun, 2004). From the positive psychology perspective, resilience is an important concept to explain performance at work and in academic environments (Salanova et al., 2009).

Links between resilience and academic achievement remain relatively scarce, but findings from studies in educational settings, such as the one carried out by Kwok et al. (2007) and Liew et al. (2018), suggest that childhood resilience has short- and long-term links to learning and achievement. Regarding studies with university students, Kwek et al. (2013) found that self-esteem and resilience are significant predictors of academic achievement. In this line, the study of Ayala and Manzano (2018) suggests that resilience and engagement should be taken into account at the time of college admission if academic achievement outcomes are sought to be improved. Maintaining resilience in educational settings may help students to reduce the presence of depression or anxiety, thus positively affecting potential academic achievement and their well-being both now and later in life (Challen et al., 2014). On the other hand, some studies do not confirm the relationship between resilience and academic achievement (Sanders and Lushington, 2002; Elizondo-Omaña et al., 2010; Sarwar et al., 2010). Finally, other studies report mixed results. Lee et al. (2012) stated that resilience is positively related to GPA (grade point average), but not to other indicators such as SAT (previously, Scholastic Assessment Test) and ACT (American College Testing), with different results for different groups of students. Allan et al. (2014) reported similar results, with mixed effects of resilience on academic achievement in United Kingdom university students. This scenario leads us to design studies taking into account other variables in order to clarify the contribution of resilience to academic performance in university students.

### Emotional Intelligence and Academic Achievement

The model proposed by Mayer and Salovey (1997) describes Emotional Intelligence as “the ability to accurately perceive, appraise, and express emotions accurately; the ability to access and/or generate feelings that facilitate thinking; the ability to understand emotion and emotional knowledge; and the ability to regulate emotions and promote emotional and intellectual development” (Mayer and Salovey, 1997, p. 10). In education, there is an increasing consensus among educators and researchers on the idea that emotional intelligence is an important skill that students must develop, both for their future well-being and for their future success in the workplace. There is evidence that emotional learning programs in educational contexts are effective (Durlak et al., 2011) and that non-cognitive constructs are powerful predictors of academic achievement (Poropat, 2009; Richardson et al., 2012). High emotional intelligence contributes to increased motivation, planning, and decision making, which positively impact academic performance (Downey et al., 2008). A recent meta-analysis conducted by MacCann et al. (2020) has shown that emotional intelligence is the third most important predictor after Intelligence and Conscientiousness in academic achievement. The authors also propose three mechanisms underlying the emotional intelligence/academic achievement link: (a) regulating academic emotions; (b) building social relationships in the school/university contexts and (c) academic content overlaps with emotional intelligence. The relationship between emotional intelligence and academic performance may be moderated by personality and self-concept. Thus, self-esteem has been found to be positively related to academic achievement (Harter, 2006; Freudenthaler et al., 2008; Pelkonen et al., 2008) and positively related to emotional intelligence (Schutte et al., 2002; Extremera and Fernández-Berrocal, 2004; Sillick and Schutte, 2006). A recent systematic review and meta-analysis undertaken by Akpur (2020) revealed that the mean effect size between emotional intelligence and academic achievement was 0.73. These findings confirm the positive impact of emotional intelligence on academic achievement.

### Self-Concept and Academic Achievement

Self-concept is a psychological construct with multiple dimensions that affect “the self’s nature of experience, including cognition, emotion, and motivation” (Markus and Kitayama, 1991, p. 224). Self-concept refers to the combination of ideas, feelings, and attitudes that people have about themselves. Likewise, it refers to the set of perceptions or reference points that the individual has about him/herself: the set of characteristics, attributes, qualities and deficiencies, capabilities and limits, values and relationships that the subject knows and perceives as data referring to his/her identity (Sánchez Moreno and Barrón López de Roda, 2007; Nalah, 2014). Kaur et al. (2009) stated that self-concept has three main elements: self-image or self-identity of an individual, self-esteem or the value that a person instills in him/herself, and the behavioral component, in which self-concept both influences and shapes a person’s behavior. Self-concept is different from self-esteem, as it is part of self-learning, predictable, and relevant to one’s own mental states and attitudes.

At present, the multidimensional character of self-concept is proven, although doubts remain as to how many factors constitute it and whether there is a relationship between the different factors. Regarding the relationship that the different factors which constitute the self-concept may have with each other, 6 different models have been described:

1. The multidimensional model of independent factors is the antithesis of the unidimensional model, given that it proposes that there is no correlation between the factors of self-concept, although a less restrictive version of it defends the relative absence of such correlation, which has received some empirical support (Soares and Soares, 1977; Marsh and Shavelson, 1985), and less so its more restrictive version (Marsh and Hattie, 1996; Marsh, 1997).
2. The multidimensional model of correlated factors assumes that all factors of self-concept are related to each other, having received much more empirical support than the model of independent factors (Marsh, 1997).
3. The multidimensional multifaceted model (Marsh and Hattie, 1996; Hattie, 2014) has a single facet (the content of the self-concept domains) that presents multiple levels, which are the different domains of self-concept (physical, social and academic).
4. The multidimensional multifaceted taxonomic model differs from the previous model, as it has at least two facets, and each of them has at least two levels (Marsh and Hattie, 1996; Hattie, 2014).
5. The compensatory model described by Winne and Marz (1981) supports the existence of a general facet of self-concept, in which the more specific facets are inversely related and integrated.
6. The multidimensional hierarchical factor model proposes that self-concept is formed by multiple dimensions organized hierarchically, where the general self-concept dominates the structure’s apex (Shavelson et al., 1976).

The hierarchical and multifaceted model of self-concept postulates that the overall self-concept has four dimensions: academic self-concept, social self-concept, emotional self-concept, and physical self-concept (Shavelson et al., 1976). Thus, self-concept in relation to academic performance is essential to an individual’s activities (Tus, 2020). Students’ college experience is strongly linked to their aspects of self-concept (Osborne and Jones, 2011); for example, their independence, belief and aspiration under the concept of personal/academic self (Rodriguez, 2009), fear of failure (the negative self), the self they want to become (the ideal self) and connection with others under the concept of the social self. The study conducted by Chamundeswari et al. (2014) found a significant correlation between self-concept, academic achievement and students’ study habits. Sikhwari (2014) investigated the relationship between motivation, self-concept and academic performance of university students. His results indicated that there was a significant correlation between motivation, self-concept and academic achievement in the sample of university students. Studies in educational contexts have found strong relationships between self-concept, academic motivation and academic achievement (Affun-Osei et al., 2014; Peixoto et al., 2016). Similarly, a meta-analysis (Huang, 2011) that analyzed the relationship between self-concept and academic achievement reported that the strength between these two constructs changed over time (Huang, 2011).

### Our Study

Specifically, this study adds to previous research aimed at analyzing the relation between resilience, emotional intelligence, self-concept and the academic achievement of university students. To this end, we propose a structural equation model in which we aim to demonstrate the moderating effect of university students’ self-concept on their academic performance. In this manner, the joint contribution of each construct in the attainment of academic achievement in university students would be studied and, according to the data found, it would allow design future training programs based on these constructs to improve academic achievement in these students.

## Materials and Methods

### Participants

The sample was constituted by 1,020 university students who were studying education degrees in Southern Spain. Regarding gender, it was found that 75.78% were women and 24.21% were men. The age of the participants ranged from 17 to 50 years (*M*: 21.52; *SD*: 4.44). Regarding the degree they studied, 42.8% were enrolled in Primary Education, 30.7% in Early Childhood Education, 14.4% in Social Education, 10.4% in a Master’s degree in Teaching and 1.7% in Pedagogy. With respect to the academic year they studied, 57.2% were enrolled in the first year, 9.9% in the second year, 18.7% in the third year and 14.2% in the fourth year. Finally, with regard to the region, 56.5% were studying in Jaén, followed by Granada (13.1%), Córdoba (10.4%), Seville (5.5%), Cádiz (5.4%), Málaga (4.2%), Almerìa (2.7%), and Huelva (2.2%).

### Instruments

#### Wong and Law Emotional Intelligence Scale

This scale (Wong and Law, 2002) is composed of 16 short statements used to evaluate four dimensions: Self-Emotion Appraisal (SEA), Other’s Emotion Appraisal (OEA), Use of Emotion (UOE), and Regulation of Emotion (ROE). Respondents are asked to rate their agreement with the statements on a five-point Likert scale ranging from 1 (strongly disagree) to 5 (strongly agree). We used the Spanish version of Extremera et al. (2019), which has shown adequate validity and reliability in Spanish contexts (α = 0.91.). This instrument has been previously used in other studies in the Spanish context (Peláez-Fernández et al., 2021; Yudes et al., 2021).

#### Resilience Scale (RS-14)

This instrument (Wagnild and Young, 1993; Wagnild, 2009, 2010) was designed to assess the extent of individual resilience through equanimity, which refers to a balanced perspective on life and experiences. Consequently, it could be seen as a person’s ability to sit back and accept what may happen, thus moderating extreme responses to adversity, which is a construct often related to sense of humor. In this study, we used the RS-14 scale validated by Sánchez-Teruel and Robles-Bello (2015) in order to determine resilience in accordance with previous studies (Sánchez-Teruel et al., 2020; Sánchez-Teruel et al., 2021). It consists of 14 items, distributed in two dimensions: (a) Personal competence and (b) Self-acceptance and life acceptance. The reliability analysis of the scale was α = 0.93.

#### Self-Concept Scale Form-5

This instrument designed by Garcìa and Musitu, (1999) has been used in previous similar studies in Spain (Suriá-Martìnez et al., 2019; Cachón-Zagalaz et al., 2020). This test measures the dimensions of academic self-concept, social self-concept, emotional self-concept, family self-concept and physical self-concept. It is composed of 30 items, which are rated with a 5-point Likert scale (Ranging from 1 = Never to 5 = Always). The total reliability of the scale was α = 0.810, and for each dimension, we found the following: academic α = 0.887; social α = 0.674; emotional α = 0.702; family α = 0.849 and physical α = 0.735 (Garcìa and Musitu, 1999).

#### Academic Achievement

The academic record was established as an objective “measure” that could be obtained and quantitatively evaluated, due to its nature and to the sample size. In this regard, the students were asked to state their average mark of their degree to date (the overall average mark obtained in the course by the student). For this purpose, they had to check their academic record and wrote down the average score that appeared at that moment.

### Procedure

In order to simplify the fulfillment of the different scales used in this study, all of them were unified in a single instrument through the Google Form tool. All the researchers attended the classes of the potential participants to explain the purpose of the research. In those cases where this was not possible, the teachers were informed to transfer the information to their students and provide them the Google Form link to complete the questionnaire. In all cases, the emails of the researchers were provided for contact in case of doubts or need for further information. Participation in the study was completely voluntary, based on the Declaration of Helsinki in 1975 and its adjustment of Brazil in 2013. In addition, the study respected the national legislation for clinical trials (223/2004 Law from February 6th), biomedical research (14/2007 Law from July 3rd) and participant’s confidentiality (15/1999 from December 13th). Furthermore, this research was approved by the Human Research Ethics Committee of the University of Jaén (code OCT.20/1.TES), regulated by Andalusian Decree 439/2010 of December 14th.

### Data Analysis

For all the analyses carried out in the study, we set an α value of 0.05. All the analyses in the study were performed with the R program. The variables treated in the study were Emotional Intelligence (EI), Resilience (Res), Self-Concept (SC) and the students’ average mark in their degree (Mark). Prior to the factorial treatment, the data were examined by data screening to analyze the necessary assumptions for the factorial treatment and their distribution. A Confirmatory Factor Analysis (CFA) was performed with each of the resulting data for each scale to verify the validity and internal consistency of these scales. CFA and SEM model analysis was conducted through the r lavaan package (Rosseel, 2012). However, due to the fact that our data were not multivariate normally distributed, the diagonally weighted least squares estimator (DWLS, Finney and DiStefano, 2013) was used. For the study of the reliability of the scales used, Cronbach’s alpha and McDonald’s ω indices were used (Revelle, 2019). Once the factorial treatment was conducted, the original scores given by the students in each questionnaire was scaled by the standardized factor loading obtained in the CFA (Beaujean, 2014). After scaling, the proposed mediational model was analyzed using structural equation-based analysis (SEM).

## Results

Mardia’s Multivariate Normality Test was performed to analyze multivariate normality. The obtained results indicated that our data did not maintain a multivariate normal distribution (Z_Kurtosis_ = 80.77, *p* < 0.01). We conducted a data screening of the data before the factor treatment to explore their distribution and analyze any assumptions. The correlation of variables to analyze additivity showed that our data did not show multicollinearity (*r* > 0.90), nor uniqueness (*r* > 0.95). In order to analyze linearity, homogeneity and homoscedasticity, we conducted a linear regression with our data and a randomly created data series. Subsequently, we explored the residuals of that regression; if there was any anomaly in the distribution of the residuals, this would be due to the distribution of our data, since the other variable was random (Kline, 2015). The distribution of the residuals did not show any anomaly, ranging most of them between –2 and + 2.

### Analysis of the Subscales

With the aim of analyzing the validity and internal consistency of the scales used in the present study, we conducted a CFA with each of the data sets obtained with each of the scales. The results of each CFA are presented below.

#### Self-Concept Scale Form 5 (AF5)

In the analysis of the Self-concept Scale Form 5 (AF5) we found that standardized factor loads varied between0.159 (SE 0.017) and0.834 (SE 0.032) (for more details, see Table 1). Then, the CFA for the AF5 scale shows an excellent fit (Hair et al., 2010), χ^2^(387) = 1,400.69, *p* < 0.001, with the following values: CFI = 0.923, TLI = 0.913, SRMR = 0.060, RMSEA = 0.051 [RMSEA 90% CI (0.048, 0.054)]. The reliability of this scale was Cronbach’s α = 0.849 and McDonald’s ω = 0.871.

**TABLE 1:** Factor loading.

Scale	Latent	Indicator	Estimate	*SE*	*Z*	*p*	Stand.
	factor						Estimate
RES	res	re1	0.593	0.018	32.326	< 0.001	0.593
		re2	0.492	0.020	24.064	< 0.001	0.492
		re3	0.377	0.016	22.862	< 0.001	0.377
		re4	0.695	0.019	37.464	< 0.001	0.695
		re5	0.719	0.019	37.901	< 0.001	0.719
		re6	0.728	0.019	38.972	< 0.001	0.728
		re7	0.493	0.017	29.496	< 0.001	0.493
		re8	0.499	0.018	27.286	< 0.001	0.499
		re9	0.447	0.018	25.536	< 0.001	0.447
		re10	0.493	0.019	26.391	< 0.001	0.493
		re11	0.701	0.018	37.958	< 0.001	0.701
		re12	0.371	0.018	20.649	< 0.001	0.371
		re13	0.527	0.019	28.026	< 0.001	0.527
EI	sea	ei1	0.702	0.023	30.157	< 0.001	0.702
		ei2	0.800	0.025	32.181	< 0.001	0.800
		ei3	0.761	0.023	32.490	< 0.001	0.761
		ei4	0.544	0.020	26.560	< 0.001	0.544
	oea	ei5	0.666	0.034	19.565	< 0.001	0.666
		ei6	0.778	0.037	20.754	< 0.001	0.778
		ei7	0.389	0.027	14.517	<0.001	0.389
		ei8	0.749	0.036	20.527	<0.001	0.749
	uoe	ei9	0.486	0.021	23.098	<0.001	0.486
		ei10	0.725	0.024	30.758	<0.001	0.725
		ei11	0.845	0.026	33.019	<0.001	0.845
		ei12	0.850	0.026	33.152	<0.001	0.850
	roe	ei13	0.722	0.022	33.002	<0.001	0.722
		ei14	0.847	0.023	36.468	<0.001	0.847
		ei15	0.578	0.020	28.825	<0.001	0.578
SC	aca	sc1	0.674	0.024	27.777	<0.001	0.674
		sc6	0.720	0.022	33.101	<0.001	0.720
		sc11	0.636	0.021	30.869	<0.001	0.636
		sc16	0.535	0.019	28.162	<0.001	0.535
		sc21	0.759	0.023	33.387	<0.001	0.759
		sc26	0.717	0.023	31.746	<0.001	0.717
	emo	sc3	0.622	0.028	22.241	<0.001	0.622
		sc8	0.704	0.03	23.779	<0.001	0.704
		sc13	0.497	0.025	20.036	<0.001	0.497
		sc18	0.375	0.026	14.696	<0.001	0.375
		sc23	0.444	0.026	16.934	<0.001	0.444
		sc28	0.533	0.026	20.238	<0.001	0.533
	fam	sc4	0.265	0.019	14.226	<0.001	0.265
		sc9	0.834	0.029	28.391	<0.001	0.834
		sc14	0.208	0.018	11.703	<0.001	0.208
		sc19	0.763	0.032	24.127	<0.001	0.763
		sc24	0.798	0.028	28.875	<0.001	0.798
		sc29	0.834	0.032	26.002	<0.001	0.834
	phy	sc5	0.543	0.02	27.855	<0.001	0.543
		sc10	0.360	0.019	19.433	<0.001	0.36
		sc15	0.579	0.02	29.401	<0.001	0.579
		sc20	0.602	0.023	26.643	<0.001	0.602
		sc25	0.442	0.02	22.262	<0.001	0.442
		sc30	0.640	0.025	25.530	<0.001	0.64
	soc	sc2	0.682	0.024	28.092	<0.001	0.682
		sc7	0.818	0.028	28.711	<0.001	0.818
		sc12	0.159	0.017	9.551	<0.001	0.159
		sc17	0.791	0.028	28.66	<0.001	0.791
		sc22	0.031	0.015	2.020	0.043	0.031
		sc27	0.630	0.022	29.021	<0.001	0.63

*RES, Resilience; SC, Self-concept; EI, Emotional intelligent; sea, Self-Emotion Appraisal; oea, Other’s Emotion Appraisal; uoe, Use of Emotion; roe, Regulation of Emotion; aca, Academic; emo, Emotional; fam, Familiar; phy, Physical; soc, Social.*

#### Resilience Scale (RS-14)

For the Resilience Scale (RS-14), the standardized factor loads ranged between0.377 (SE 0.016) and0.728 (SE 0.019) (for more details regarding the internal consistency, see Table 1). The CFA for the RS-14 scale indicates an excellent fit (Hair et al., 2010), χ^2^(77) = 279.935, *p* < 0.001, with CFI = 0.972, TLI = 0.967, SRMR = 0.063, RMSEA = 0.051 [RMSEA 90% CI (0.045, 0.057)]. In addition, the reliability of this scale was Cronbach’s α = 0.867 and McDonald’s ω = 0.868.

#### Wong Law Emotional Intelligence Scale

In the case of the WLEIS-S, the standardized factor loads ranged between0.389 (SE 0.027) and0.890 (SE 0.024). The complete data on internal consistency are available in Table 1. The CFA for the WLEIS-S scale shows an excellent fit (Hair et al., 2010), χ^2^(98) = 183.180, *p* < 0.001, with CFI = 0.989, TLI = 0.987, SRMR = 0.041, RMSEA = 0.029 (RMSEA 90% CI (0.023, 0.036)]. The reliability of this scale was Cronbach’s α = 0.834 and McDonald’s ω = 0.894.

### Mediational Analysis

**FIGURE 1:**
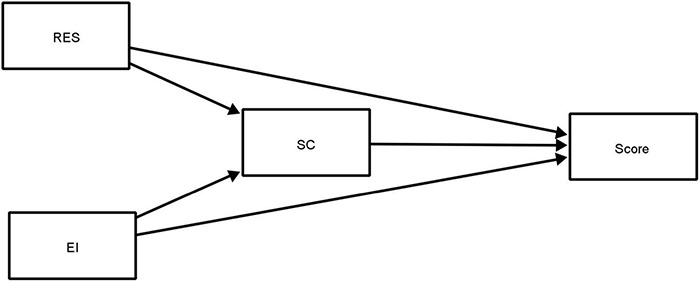
Theoretical mediational model.

**FIGURE 2:**
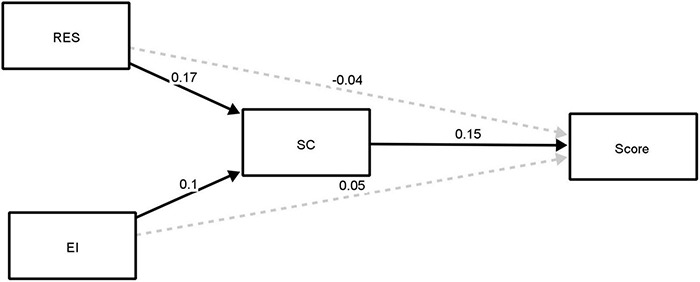
Mediational model results.

Figure 1 displays the proposed mediation model that the study sought to analyze. Within this figure, the squares represent the values of the scaled variables obtained from each of the scales. The one-way arrows indicates regression relationships. Table 2 shows the results of the analysis for both direct and indirect regression relationships in the mediational model. Figure 2 shows the summarized results of the proposed model. The continuous black arrows show the significant relationships, while the dashed gray arrows show the non-significant relationships of the model. As can be observed, all significant relationships in the model involve self-concept. Thus, the only factor that is significantly directly related to the mark is self-concept (β = 0.15, *p* < 0.001). That is, those students with a higher self-concept will show better academic performance. Additionally, self-concept indirectly mediates the relationship between resilience and Mark (β = 0.02, *p* = 0.004), and the indirect mediating relationship between emotional intelligence and mark is very close to significance (β = 0.01 *p* = 0.056). The remaining significant relationships were between self-concept and resilience (β = 0.17, *p* > 0.001) and between self-concept and emotional intelligence (β = 0.10, *p* = 0.035).

**TABLE 2:** Indirect and total effects on mediational analysis.

				95% C.I.				
Type	Effect	Estimate	SE	Lower	Upper	β	*z*	*P*
Indirect	RES ⇒ SC ⇒ Note	0.06	0.02	0.02	0.10	0.02	2.86	0.004
	EI ⇒ SC ⇒ Note	0.05	0.02	–0.00	0.09	0.01	1.91	0.056
Component	RES ⇒ SC	0.10	0.03	0.05	0.16	0.17	3.69	<0.001
	SC ⇒ Note	0.59	0.13	0.33	0.84	0.15	4.54	<0.001
	EI ⇒ SC	0.08	0.04	0.01	0.15	0.10	2.11	0.035
Direct	RES ⇒ Note	–0.09	0.12	–0.33	0.14	–0.04	–0.80	0.423
	EI ⇒ Note	0.15	0.15	–0.15	0.44	0.05	0.98	0.328
Total	RES ⇒ Note	–0.03	0.12	–0.27	0.20	–0.01	–0.28	0.780
	EI ⇒ Note	0.19	0.15	–0.11	0.49	0.06	1.27	0.205

*RES, Resilience; SC, Self-concept; EI, Emotional intelligent; Note, Academic record.*

These results suggest that, although resilience or emotional intelligence are not able to predict students’ academic scores directly, they are able to do it through self-concept. Thus, students with high emotional intelligence or resilience and who also show high levels of self-concept will predict high academic grade scores.

Previous studies have shown that the sex variable may unequally affect the relationships between some of the variables in the model. In such case, part of the effects shown in the model could be due to this unequal modulating effect of the sex variable. In order to control for this, the analysis of the model was carried out, but this time the sex variable was introduced as a modulator of the relationships in the model. The results of the analysis indicate that the sex variable did not show a significant moderating effect on any of the relationships proposed in the model (greater effect EI: Sex ⇒ SC β = 0.091 *p* = 0.089).

## Discussion and Conclusion

The present study analyzed the relationship between resilience, emotional intelligence and self-concept on academic achievement in university students. Our study shows that there is no direct relationship between resilience and academic achievement, nor between emotional intelligence and academic achievement. These results differ from those found in other studies. In this regard, a recent meta-analysis by MacCann et al. (2020) pointed out that emotional intelligence is the third most influential factor on academic achievement. Among their findings, they reported that self-assessed emotional intelligence was a stronger predictor of grades than standardized test scores. On the other hand, the research conducted by Haktanir et al. (2021) with university population, whose purpose was to examine the role of psychological constructs on adaptation in first-year students, found significant direct relationships between resilience and academic self-concept, as well as between resilience and university adaptation. Similarly, significant relationships were found between self-concept and college adaptation. In our case, a significant relationship was also found between resilience, self-concept and academic achievement. In other words, self-concept mediates the relationship between resilience and academic achievement. Previous studies have indicated that the self-concept of ability, for instance, the beliefs that students have about their academic performance in different areas of knowledge, are related to their academic achievement (Susperreguy et al., 2018). No significant mediation results were found between emotional intelligence, self-concept and academic achievement. From these data, it is clear that university students who exhibit high levels of resilience tend to cope better with difficult times and understand and value effort. Other studies have also reported the important mediating role of resilience (Garcìa-Martìnez et al., in press) in the mental health and personality factors of college students. This study supports positive beliefs and behaviors for better academic achievement. In this regard, resilient students will be more likely to cope with contextual demands, especially those related to the university setting, and this attitude will determine their academic success. These findings are in line with those found by Hartley (2011), who highlights the importance of resilience in academic achievement. According to previous research (Haktanir et al., 2021) resilience is a significant predictor of self-concept. In this study, our findings revealed that students with higher resilience and self-concept show better academic performance. In this same vein, the data found are in line with the meta-analysis conducted by Huang (2011), where it was found that a high self-concept is related to high academic achievement. Moreover, to some extent, these factors affect students’ academic performance. Similarly, the literature suggests that emotional intelligence is directly related to students’ academic achievement and indirectly related to academic stress (Garcìa-Martìnez et al., 2021). Likewise, emotional intelligence is related to students’ educational engagement, which, in turn, promotes the attainment of greater academic success among students (Mérida-López et al., 2021).

The fact that the relationship between emotional intelligence and academic achievement is not linear and direct (as it appears in this study) could be due to the influence of other individual characteristics or variables of the students. In a study conducted by Fernández-Berrocal et al. (2003) with high school students, they found connections between school performance and emotional intelligence; specifically, it was found that intrapersonal emotional intelligence influences students’ mental health and this psychological balance, in turn, is related to and affects final academic performance. This finding is consistent with the results of previous research, which confirm that individuals with certain deficits (e.g., poor skills, emotional maladjustment, learning disabilities, etc.,) are more likely to experience stress and emotional difficulties during their studies and, consequently, would benefit more from the use of adaptive emotional skills that allow them to cope with such difficulties.

Regarding advances in the field of study, studies on understanding relationships between emotional intelligence, resilience and self-concept in predicting academic achievement components may be beneficial, on the one hand, for students who are inclined toward disciplines that are more likely to succeed. On the other hand, it is also useful for researchers interested in the determining factors of academic success, to address the weaknesses of students coming into the classroom and cultivating the strengths that may help them to perform better academically. Anxiety, stress and emotional deficits are some of the factors that may negatively influence academic achievement, and high emotional intelligence, as well as good resilience, could have a major effect when the demands of a particular situation tend to overwhelm students’ intellectual resources.

### Practical Implications

In view of the results obtained, it would be advisable to develop and implement intervention programs to help university students with low performance to participate in these programs that develop strategies based on resilience, in order to directly affect the development of self-concept, which may thereby improve the academic development of students. These intervention programs should combine self-improvement and the developmental ability of the individuals should be integrated (Huang, 2011). Several resilience training programs have been put into practice; for instance, Sternberg (2003) proposed the training program called “Another 3R.” This program focuses on personal interaction with the environment and how to solve individual issues effectively, and it requires students to learn about reasoning, develop resilience, and be more responsible. Currently, the Resilience Program “Pennsylvania Resilience Program (PRP)” developed by Seligman (2003), is a training program based on cognitive-behavioral theory that focuses on improving students’ behavior and cognitive skills (Kumpfer, 1999).

### Limitations

This research is not without limitations. Firstly, the design of this study is cross-sectional. This design means that no causal effect can be established between the study variables. Further studies will have to take into account mediational models through a longitudinal design involving students from the first year to the end of their degree to determine the magnitude and direction of the changes experienced by these students. In addition, there is the limitation regarding the study sample, since the proportion of men and women is unequal. However, this proportion is consistent with that reported by the Spanish Institute of Statistics for university population in Education degrees (Spanish Institute of Statistics, 2020).

Furthermore, all the data collection instruments used to assess the psychological constructs analyzed in the study are based on self-report measures. Future research should consider the use of ability measures (Mayer et al., 2003). As for the measurement of academic performance, students were asked to indicate their current average grade, which may be a non-objective measure, as students may misreport this datum. An objective achievement test could have overcome this limitation, but the sample size and the conditions under which the instruments were administered did not make this possible.

## Data Availability Statement

The datasets generated during and/or analyzed during the current study are available from the corresponding author on reasonable request.

## Ethics Statement

This research is approved by the Human Research Ethics Committee of the University of Jaén (code OCT.20/1.TES). The patients/participants provided their written informed consent to participate in this study.

## Author Contributions

SL, JA-L, RQ-L, and IG-M: conceptualization and supervision. SL and JA-L: methodology. SL: software. RQ-L and IG-M: writing—original draft preparation. SL, JA-L, and IG-M: writing—review and editing. All authors have read and agreed to the published version of the manuscript.

## Conflict of Interest

The authors declare that the research was conducted in the absence of any commercial or financial relationships that could be construed as a potential conflict of interest. The reviewer MA-L declared a shared affiliation with one of the authors, IG-M, to the handling editor at the time of the review.

## Publisher’s Note

All claims expressed in this article are solely those of the authors and do not necessarily represent those of their affiliated organizations, or those of the publisher, the editors and the reviewers. Any product that may be evaluated in this article, or claim that may be made by its manufacturer, is not guaranteed or endorsed by the publisher.
